# The ChEMBL bioactivity database: an update

**DOI:** 10.1093/nar/gkt1031

**Published:** 2013-11-07

**Authors:** A. Patrícia Bento, Anna Gaulton, Anne Hersey, Louisa J. Bellis, Jon Chambers, Mark Davies, Felix A. Krüger, Yvonne Light, Lora Mak, Shaun McGlinchey, Michal Nowotka, George Papadatos, Rita Santos, John P. Overington

**Affiliations:** European Molecular Biology Laboratory European Bioinformatics Institute, Wellcome Trust Genome Campus, Hinxton, Cambridgeshire CB10 1SD, UK

## Abstract

ChEMBL is an open large-scale bioactivity database (https://www.ebi.ac.uk/chembl), previously described in the 2012 Nucleic Acids Research Database Issue. Since then, a variety of new data sources and improvements in functionality have contributed to the growth and utility of the resource. In particular, more comprehensive tracking of compounds from research stages through clinical development to market is provided through the inclusion of data from United States Adopted Name applications; a new richer data model for representing drug targets has been developed; and a number of methods have been put in place to allow users to more easily identify reliable data. Finally, access to ChEMBL is now available via a new Resource Description Framework format, in addition to the web-based interface, data downloads and web services.

## INTRODUCTION

ChEMBL is an open large-scale bioactivity database containing information largely manually extracted from the medicinal chemistry literature. Information regarding the compounds tested (including their structures), the biological or physicochemical assays performed on these and the targets of these assays are recorded in a structured form, allowing users to address a broad range of drug discovery questions. Applications of the data include the identification of suitable chemical tools for a target; investigation of the selectivity and off-targets effects of drugs; large-scale data mining, such as the construction of predictive models for targets and identification of bioisostere replacements or activity cliffs (1–4); and as a key component of integrated drug discovery platforms (5–7). In addition to literature-extracted information, ChEMBL also integrates deposited screening results and bioactivity data from other key public databases [e.g. PubChem BioAssay (8)], and information about approved drugs from resources such as the U.S. Food and Drug Administration (FDA) Orange Book (9) and DailyMed (http://dailymed.nlm.nih.gov/dailymed). Details of the data extraction process, curation and data model have been published previously (10); therefore, the current article focuses on recent enhancements to ChEMBL.

## DATA CONTENT

Release 17 of the ChEMBL database contains information extracted from >51 000 publications, together with bioactivity data sets from 18 other sources (depositors and databases). In total, there are now >1.3 million distinct compound structures and 12 million bioactivity data points. The data are mapped to >9000 targets, of which 2827 are human protein targets. Data sets added over the past 2 years include the following: neglected disease screening results from projects funded by Medicines for Malaria Venture (11), Drugs for Neglected Diseases initiative (http://www.dndi.org), World Health Organization TDR programme (WHO-TDR) (12), Open Source Malaria (http://opensourcemalaria.org), Harvard University (13) and GlaxoSmithKline (14); kinase screening results from Millipore (15), and several groups using the Protein Kinase Inhibitor Set compound collection (16); supplementary bioactivity data associated with publications from GlaxoSmithKline (17–19); and information from several other databases including DrugMatrix (https://ntp.niehs.nih.gov/drugmatrix/index.html), TP-search (20) and Open TG-GATEs (21).

## NEW DEVELOPMENTS

### Tracking compound progression

**Figure 1.**
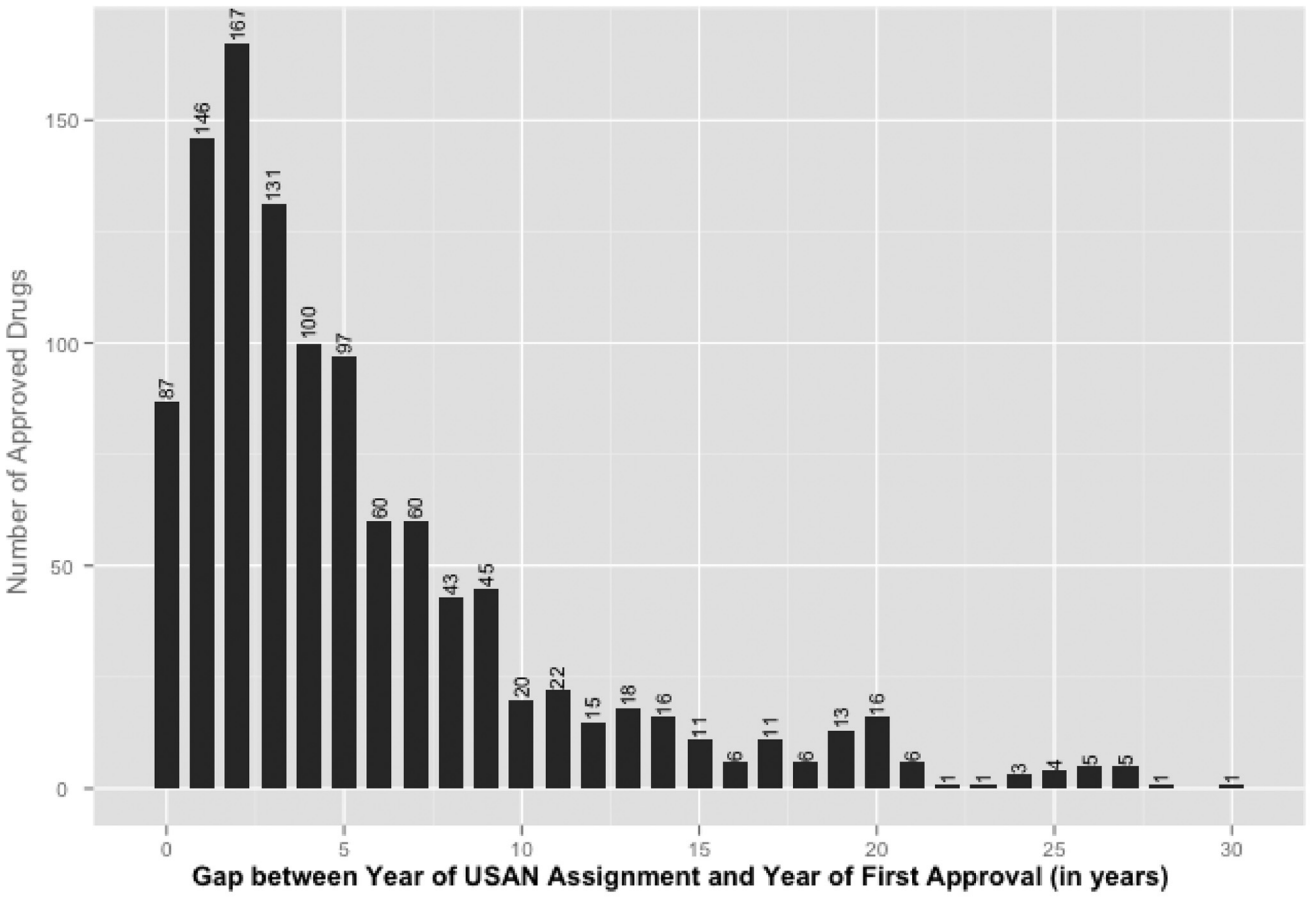
Frequency distribution for approved drugs, showing the number of years taken for a drug to be approved after a USAN was assigned to it.

Although the extraction of structure–activity relationship data from medicinal chemistry literature provides a good overview of drug discovery research, a fuller picture of drugs in development and marketed products is obtained only by combining literature data with other information sources. To increase the coverage of drugs in development (to complement the set of approved drugs already included in ChEMBL from the FDA Orange Book), we have now added structures and annotation for >10 000 compounds and biotherapeutics for which United States Adopted Name (USAN) or International Nonproprietary Name (INN) applications have been filed. This information has been obtained from the public list of adopted names provided by the USANs Council (http://www.ama-assn.org/ama/pub/physician-resources/medical-science/united-states-adopted-names-council/adopted-names.page) and the USP dictionary of USAN and International Drug Names (22). The application for a USAN or INN is typically made when a compound is in early/mid-stage development and therefore serves as a robust general overview of clinical candidate space. Structures for novel candidates are manually assigned and, for protein therapeutics, amino acid sequences may be annotated, where available. For each parent compound, information regarding its synonyms, research codes, applicants, year of USAN assignment and the indication class for which the USAN has been initially filed, where available, is also included in the database. The synonyms consist of the non-proprietary names for the compounds containing that parent molecule, and respective type (or source) of that name, such as the FDA name, USAN, INN, British Approved Name (BAN), Japanese Accepted Name (JAN) and French approved non-proprietary name (Dénomination Commune Française, DCF). The inclusion of research codes and synonyms from multiple sources maximizes the chance of finding a compound of interest based on text searches, and allows adaptive searching across the literature, reflecting the changing names of compounds as they are cross-licensed and/or progress to later clinical stages. The year of USAN assignment can be used to roughly infer the likelihood of a compound being approved. Typically, an approved drug gets its USAN assigned between 1–3 years before approval, and only a small fraction of drugs is approved when the USAN is 10 years or older (see Figure 1).

**Figure 2.**
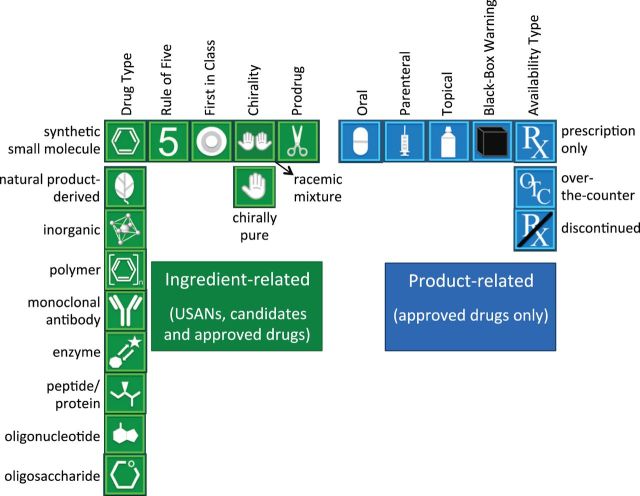
For rapid visual comparison, the ChEMBL interface displays a set of icons that summarize features of the chemical compound/ingredient (green icons) as well as any marketed products (blue icons).

For each compound, ChEMBL also provides the USAN or INN stems assigned by the USANs council or the WHO, respectively. These are prefixes, suffixes and infixes in the non-proprietary name, which emphasize a specific chemical structure type, a pharmacological property/mechanism of action or a combination of these. In addition to the USAN-derived information, each of the compounds is also annotated with its respective Anatomical Therapeutic Chemical (ATC) code, where available. The ATC classification is assigned by the WHO Collaborating Center for Drug Statistics Methodology (23) and can be used as a tool for comparing data on drugs regarding the organ or anatomical system on which they act and their therapeutic, pharmacological and chemical profile. ChEMBL also provides users with a rapid assessment of the important compound/ingredient features, such as drug type (synthetic small molecule, natural product-derived, inorganic, polymer, antibody, peptide/protein, oligonucleotide or oligosaccharide), whether the compound violates any of the Rule-of-Five criteria, whether it exerts its pharmacological action by a novel mechanism, whether it is dosed as a defined single stereoisomer or racemic mixture and whether it is a known prodrug (see Figure 2).

The annotation of the compounds in preclinical research and development serves as a platform for the annotation of the FDA-approved drugs. It is likely that, when a novel drug is finally marketed, much the information regarding the active molecule can already be found in ChEMBL. For each launched drug, the ingredient-derived data are then complemented with information regarding its marketed product. This includes information regarding its trade names, dosage information, approval dates, administration routes, whether there are ‘black box’ safety warnings associated with the product, whether it has a therapeutic application (as opposed to imaging/diagnostic agents, additives etc) and finally whether the product is available on prescription, over-the-counter or, if eventually, it has been discontinued. This information allows users of the bioactivity data to assess whether a compound of interest is an approved drug and is, therefore, likely to have an advantageous safety/pharmacokinetic profile or be orally bioavailable, for example.

Finally, FDA-approved and WHO anti-malarial drugs have now been annotated with mechanism of action and efficacy target information. This information has been manually assigned, using primary sources such as published literature and manufacturer’s prescribing information. Targets have only been included for a drug if (i) the drug is believed to interact directly with the target; and (ii) there is evidence that this interaction contributes toward the efficacy of that drug in the indication(s) for which it is approved. We do not currently list as drug targets proteins that are responsible for the pharmacokinetics or adverse effects of a drug, those for which there is pharmacology data but no known link to *in vivo* efficacy (though these can obviously be queried from the bioactivity data in ChEMBL), or those that may be relevant to other indications for which the drug is not currently approved.

### Modeling drug targets

The appropriate representation of drug targets in bioactivity databases is a non-trivial issue. In certain circumstances it may be sufficient to consider a ‘target’ and a ‘protein’ as synonymous. However, there are numerous cases where this oversimplification breaks down. A large number of marketed drugs, for example, bind to protein complexes or bind non-selectively to all members of a protein family. Similarly, for published assay data, if a measurement has been performed in a cell-based assay it is often not clear which of several related proteins are responsible for the effect observed. Formerly assays in ChEMBL that described the interaction of a compound with multiple possible proteins were mapped to several targets. However, this representation was suboptimal for a number of reasons. Firstly, users might have retrieved multiple rows of data for a compound of interest and erroneously believed that it has been tested in multiple different assays against each of the individual targets. In addition, a user querying the database with a non–compound-binding subunit of a protein complex would still retrieve activity data and, again, might incorrectly infer that compounds identified bind directly to that subunit. A new target data model has, therefore, been developed within ChEMBL to draw a clear distinction between targets (the entities with which a compound interacts to exert its effects) and the molecular components (usually proteins) that make up those targets.

A key step in the development of the new data model was the definition of new target types. The original ‘PROTEIN’ target type has now been subdivided into a number of categories. In the simple case where a compound is believed to interact specifically with a monomeric protein, the target type ‘SINGLE PROTEIN’ is now used. In cases where either a compound is known to act non-specifically with all members of a protein family, or the assay conditions are such that it is not possible to determine which member(s) of a protein family the compound is acting on (e.g. a cell-based or tissue-based assay), a target type of ‘PROTEIN FAMILY’ is used. The target represents, and is linked to, the group of all proteins (components) with which the compound may interact. Where the molecular entity with which the compound interacts is known to be a protein complex, and can be precisely defined, the target type ‘PROTEIN COMPLEX’ is used. Again, the target represents the complex itself, but is linked to components representing each of the protein subunits. However, it is not always possible to define protein complex targets precisely. For example, compounds are often measured for activity at gamma-aminobutyric acid (GABA-A) receptors in tissue-based assays. Although GABA-A receptors are known to be pentameric ligand-gated ion channels, they can consist of various combinations of *α*, *β* and *γ* subunits (of which there are 12 in total). In a tissue-based format, the exact subunit combinations present are generally not known. In such cases, the target type of ‘PROTEIN COMPLEX GROUP’ is used. Other new target types have also been created for approved drugs whose molecular targets are not proteins (e.g. metal chelating agents, ribosome inhibitors, antisense RNA agents). Table 1 shows a full list of all molecular target types in ChEMBL and the number of targets in each category.

**Table 1.** List of molecular target types included in release 17 of the ChEMBL database, with a description and example of each type, and the total number of targets of that type

Target type	Description	Example	Number of targets
Single protein	Single protein chain	Phosphodiesterase 5A (CHEMBL1827)	5518
Protein family	Group of closely related proteins	Muscarinic receptors (CHEMBL2094109)	188
Protein complex	Defined protein complex, consisting of multiple subunits	GABA-A receptor alpha-3/beta-3/gamma-2 (CHEMBL2094120)	159
Protein complex group	Poorly defined protein complex where subunit composition is unclear	GABA-A receptor (CHEMBL2093872)	43
Protein–protein interaction	Disruption of a protein–protein interaction	p53/Mdm2 (CHEMBL1907611)	12
Chimeric protein	Fusion of two different proteins, either a synthetic construct or naturally occurring	Bcr/Abl fusion protein (CHEMBL2096618)	2
Selectivity group	Pair of proteins for which selectivity has been assessed	Muscarinic receptors M2 and M3 (CHEMBL2095187)	96
Protein–nucleic acid complex	Complex consisting of both protein and nucleic acid components	70S ribosome (CHEMBL2363965)	6
Nucleic acid	DNA, RNA or PNA	Apo-B 100 mRNA (CHEMBL2364185)	28
Oligosaccharide	Oligosaccharide	Heparin (CHEMBL2364712)	4
Small molecule	Small molecule, such as amino acid, sugar or metabolite	Glutamine (CHEMBL2366039)	20
Macromolecule	Large biological molecule other than protein complex	Hemozoin (CHEMBL613898)	4
Metal	Metal or ion	Iron (CHEMBL2363058)	8

The new data model also allows the annotation of binding site information on protein targets. Binding sites can be defined at varying levels of granularity (subunit-level, protein domain-level or residue-level), according to the information available. This facility has been used to annotate bioactivity results with the predicted Pfam domain to which the compound is most likely to bind (24) and to annotate drug efficacy targets with known binding subunit information. For example, benzodiazepine drugs are known to bind to GABA-A receptors at the interface of α and γ subunits, but only in α-1, α-2, α-3 and α-5 containing receptors. Therefore while the ChEMBL target for these drugs contains all α, β and γ subunits, the benzodiazepine binding site definition for this target consists of only the α-1, α-2, α-3, α-5 and γ subunits. Users can therefore use this information to exclude protein subunits that are not directly involved in the binding of the drug to its target (in this case the β subunits). Full details of the latest ChEMBL data model can be seen in Supplementary Figure S1.

### Allowing users to pinpoint high-quality data

As the volume of data in ChEMBL grows, it becomes increasingly important to empower users with the ability to evaluate the quality and appropriateness of these data for their particular use cases. For example, while a researcher investigating a single target or compound series may prefer to retrieve as much data as possible, and validate this information by referring back to the original publications, for other applications, such as the training of computational models, it may be vital to exclude any data that could potentially be erroneous. Therefore, a number of different enhancements have been made to the database to allow users to more easily assess the drug-likeness of compounds, compare bioactivity values from different assays and highlight possible errors or duplications in the data.

To help users assess the drug-likeness of compounds, a set of physicochemical properties is provided for the ChEMBL compounds. The calculations are made on the parent form of the molecule, after any salts have been removed, and where the molecular weight of the compound is <1000 and the structure is comprised only of standard common atoms (C, N, O, H, F, Cl, Br, I, S, P). The exception is the full molecular weight (FULL_MWT), which, where applicable, is the molecular weight of the salt plus any hydrates present. The properties are calculated either using algorithms provided in Pipeline Pilot (version 8.5, Accelrys Inc. 2012) or using the ACDlabs Physchem software (version 12.01, Advanced Chemistry Development Inc. 2010). Some further descriptors have been derived from these properties such as the well-used Lipinski Rule-of-Five (25); the Rule-of-Three passes, used to identify compounds suitable for fragment screening (26); and the weighted Quantitative Estimate of Drug likeness (QED_WEIGHTED), for which values range from 0–1 [1 being the most drug-like and 0 the least drug-like (27)].

Ligand efficiencies are also increasingly used to identify not just compounds that have high affinity for a target but those that give maximum binding for their size, number of atoms or lipophilicity/polar atoms. There are a number of different measures now described in the literature and four of the more common have now been made available in the database: Ligand Efficiency (LE) (28), Binding Efficiency Index (BEI), Surface Efficiency Index (SEI) (29) and Lipophilic Ligand Efficiency (LLE) (30). The ligand efficiencies are calculated on the standardized pChEMBL values (see later in the text) for binding data to protein targets. For the BEI and SEI, it is also possible to see a plot of these for a specific target on the Target Report Card on the ChEMBL Web site and interactively look at the structures and select sets of the most ligand efficient molecules that bind to a target of interest. 

When identifying compounds that bind to a particular protein target for structure–activity relationship or lead identification studies, it is important to be using comparable data. We have, therefore, standardized many of the activity types and their corresponding units. For example, IC50, IC50_mean, IC50_µM and mean IC50 have all been given the standard activity type of IC50, and units of µM, mM, nmol/l and 10^−^^4 ^mol/l and so forth have been standardized to nM. Additionally a number of activities are reported in articles as the -log values (e.g. pKi, pIC50, -logIC50), we have anti-logged these values so that all of the IC50 values (whether reported in the original article as IC50 or pIC50) are seen as IC50, and hence all similar activity types can be readily identified and their values more easily compared.

In addition to the conversion of published activity types/values/units to standard activity types/values/units, an additional field called pCHEMBL_VALUE has been added to the activities table. This value allows a number of roughly comparable measures of half-maximal response concentration/potency/affinity to be compared on a negative logarithmic scale (e.g. an IC50 measurement of 1 nM has a pChEMBL value of 9). The pChEMBL value is currently defined as follows: −log_10_ (molar IC50, XC50, EC50, AC50, Ki, Kd or Potency).

Having standardized the activity types, units and values, it is also then possible to identify data that are potentially erroneous and require further checking with reference to the original article. These data are flagged in the DATA_VALIDITY_COMMENT column of the activities table, the values of which should be self-explanatory. The key ones are ‘Outside typical range’ where the value is what would normally be considered too high or low a value for the activity type (e.g. an IC50 value of 10^9 ^nM) and ‘Non standard unit for type’ where the unit is inappropriate for the activity type (e.g. an IC50 with units of % or µg). A table is available in the database (ACTIVITY_STDS_LOOKUP) that contains details of the activity types that have been standardized, their permitted standardized units and their acceptable value ranges.

Lastly the standardization of activity allows the identification of potential duplicate activity values using the rules outlined by Kramer *et al.* (31). These are values where an activity measurement reported in an article is likely to be a repeat citation of an earlier measurement, rather than an independent measurement. A particular example would be a value reported on a compound used as a standard in an assay. We flag all data where the pChEMBL value between the earliest reference and later references is <0.02 (for the same compound and target pair) with a value of 1 in the POTENTIAL_DUPLICATE column of the activities table. Similarly, wherever the two pChEMBL values differ by exactly 3 or 6 log units, the activity record from the most recent publication is flagged as a ‘Potential transcription error’.

## DATA ACCESS

### The ChEMBL interface

The ChEMBL database is accessible via a simple web-based interface at https://www.ebi.ac.uk/chembl. This interface allows users to search the database in a number of ways. A simple key word search box at the top of the page allows users to find compounds, targets, assays or documents containing a search term of interest (by searching various name, description and synonym fields). For users wishing to search for compounds by structure, rather than name, the ‘Ligand Search’ tab provides a simple sketcher, allowing users to draw a structure or substructure of interest (or import a molfile) and retrieve related compounds (32). Alternatively, compounds can also be retrieved by CHEMBL_ID, smiles or a sequence search against biotherapeutic drugs. Similarly, a ‘Target Search’ tab allows searching of targets by CHEMBL_ID or sequence. Targets can also be browsed either by organism or protein family via the ‘Browse Targets’ tab. Enhancements have recently been made to this tree browser, allowing users to search by key word and identify relevant nodes of the tree, and to expand, collapse or select multiple nodes simultaneously.

Having identified compounds or targets of interest, bioactivity data can be retrieved either from a drop-down menu on the search results pages, or via the Report Cards provided for each ChEMBL compound, target, assay or document, which contain a series of clickable graphical widgets for this purpose.

**Figure 3.**
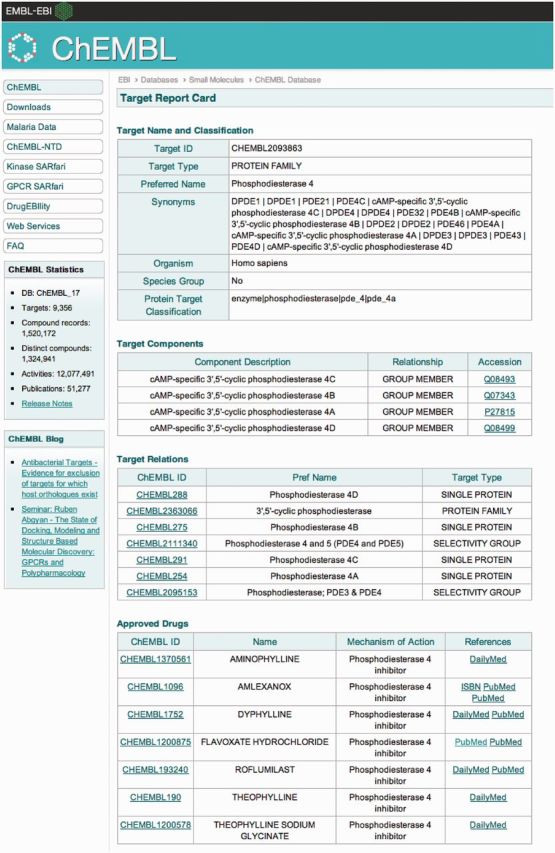
Screen capture showing enhancements to the ChEMBL Target Report Card. The Target Components section shows which proteins are components of this target (in this case members of the protein family), whereas the Target Relations section shows other targets that are related to this one because they share one or more of those components. The Approved Drugs section shows that there are approved products that are believed to exert at least part of their efficacy through inhibition of phosphodiesterase 4.

Compound and Target report card pages have been improved to incorporate more extensive cross-references to other resources. For compounds, these are now provided by the UniChem service (33), whereas for protein targets cross-references are derived either from UniProt (34) or through manual annotation. A new section on the Compound and Target Report Cards also provides mechanism of action information, where available, for approved drugs and their efficacy targets. Following the modification of the ChEMBL target data model, Target Report Cards now provide details of the protein components of each target (e.g. in the case of a protein complex or protein family) in the ‘Target Components’ section and a list of related targets (based on overlap of protein components) in the ‘Target Relations’ section. This information allows users to rapidly understand the composition of the target they are viewing and identify any other similar targets that may have relevant bioactivity data (see Figure 3).

A new feature of the Document Report Card is a section that lists other publications in ChEMBL that are deemed similar to the one featured in the Report Card. Several methods may be used to assess pairwise document similarity, e.g. overlap of Medical Subject Headings (MeSH) terms (http://www.nlm.nih.gov/mesh/) or document clustering based on a term vector approach (35). In our case, however, the similarity between two documents consists of components: the first one is defined by whether a document cites or is referenced by the other using information retrieved from EuropePMC (36), via the available web services. Having established pairs of related documents, the second component is defined by the amount of overlap between the compounds and biological targets reported in those documents, quantified by the Tanimoto coefficient (37). For example, two articles reporting assay results for the same set of compounds will have a Tanimoto score of 1, as will two documents that report assay results for the same set of targets. The documents with the highest compound and target Tanimoto similarity scores to the query document are listed in the Related Documents section (see Supplementary Figure S2).

In addition to the search tabs and report card pages, a number of tabs show different views of drug data within the database. The ‘Browse Drugs’ tab lists FDA-approved drugs and compounds from the USP Dictionary/USAN documents together with their various properties and icons (as described in Figure 2). The new ‘Browse Drug Targets’ tab lists mechanism of action information for all FDA-approved drugs and WHO anti-malarial drugs with links to the relevant Compound and Target Report Card pages and references. Finally the ‘Drug Approvals’ tab shows the most recently approved FDA drugs with links to more detailed drug monographs.

### Downloads and web services

Although the ChEMBL interface provides the basic functionality required for many common queries, some users may prefer to either download the entire database and use it locally (particularly for use in large-scale data mining or integration with other data sources) or retrieve data programmatically via web services.

The semantic web is becoming an increasingly popular platform for large-scale data integration, with many now choosing to use triple stores and storing data in Resource Description Framework (RDF) format, in preference to building traditional data warehouses. In response to demand from our users, an RDF version of ChEMBL has now been developed and is available for download from our FTP site (ftp://ftp.ebi.ac.uk/pub/databases/chembl/). The RDF model follows fairly closely the relational model and uses a basic internal ontology known as the ChEMBL Core Ontology to describe the core concepts and relationships between them. In addition, multiple external ontologies are used including BioAssay Ontology (38), Unit Ontology (39), Quantities, Units, Dimensions and Data Types Ontology (QUDT, http://qudt.org) and Chemical Information Ontology (CHEMINF) (40). A SPARQL endpoint and Linked Data browser (http://www.ebi.ac.uk/fgpt/sw/lodestar/) are also provided, allowing users to query and navigate the data: https://www.ebi.ac.uk/rdf/services/chembl/sparql.

Each release of ChEMBL is also freely available from our FTP site in a variety of other formats including Oracle, MySQL, PostGRES, a structure-data file (SDF) of compound structures and a FASTA format file of the target sequences, under a Creative Commons Attribution-ShareAlike 3.0 Unported license (http://creativecommons.org/licenses/by-sa/3.0). 

Finally, a set of Representational State Transfer (REST) based web services is also provided (together with sample Java, Perl and Python clients), to allow programmatic retrieval of ChEMBL data in extensible mark-up language or JavaScript Object Notation (JSON) formats (see https://www.ebi.ac.uk/chembl/ws for more details). 

## SUPPLEMENTARY DATA

Supplementary Data are available at NAR online.

## FUNDING

Strategic Award for Chemogenomics from the Wellcome Trust [WT086151/Z/08/Z]; European Molecular Biology Laboratory; the Innovative Medicines Initiative Joint Undertaking [115191, 115002]; Medicines for Malaria Venture. Funding for open access charge: European Molecular Biology Laboratory.

*Conflict of interest statement*. None declared.
